# Foraging habitat choice of White-tailed Tropicbirds revealed by fine-scale GPS tracking and remote sensing

**DOI:** 10.7717/peerj.6261

**Published:** 2019-01-16

**Authors:** Carlos D. Santos, Leila F.A.S. Campos, Márcio A. Efe

**Affiliations:** 1 Núcleo de Teoria e Pesquisa do Comportamento, Universidade Federal do Pará, Belém, Brazil; 2 Department of Migration and Immuno-Ecology, Max Planck Institute for Ornithology, Radolfzell, Germany; 3 Laboratório de Bioecologia e Conservação de Aves Neotropicais, Instituto de Ciências Biológicas e da Saúde, Universidade Federal de Alagoas, Maceió, Brazil

**Keywords:** Tropical seabirds, Animal tracking, Ocean productivity, MODIS, Fernando de Noronha, Oceanographic variables

## Abstract

**Background:**

The introduction of animal tracking technology has rapidly advanced our understanding of seabird foraging ecology. Tracking data is particularly powerful when combined with oceanographic information derived from satellite remote sensing, allowing insights into the functional mechanisms of marine ecosystems. While this framework has been used extensively over the last two decades, there are still vast ocean regions and many seabird species for which information is scarce, particularly in tropical oceans.

**Methods:**

In this study we tracked the movement at high GPS recording frequency of 15 White-tailed Tropicbirds (*Phaethon lepturus*) during chick-rearing from a colony in Fernando de Noronha (offshore of Northeast Brazil). Flight behaviours of travelling and searching for food were derived from GPS data and examined in relation to satellite-sensed oceanographic variables (sea surface temperature, turbidity and chlorophyll-a concentration).

**Results:**

White-tailed Tropicbirds showed marked preference for clear and warm sea surface waters, which are indicative of low primary productivity but are likely the best habitat for preying upon flying fish.

**Discussion:**

These findings are consistent with previous studies showing that foraging habitat choices of tropical seabirds may not be driven by primary productivity, as has been widely shown for non-tropical species.

## Introduction

The way how pelagic seabirds move across the vastness of the open ocean have fascinated generations of scientists, but only recently have technological developments provided the tools to uncover that mystery. Tracking devices have become indispensable tools to study the behaviour of seabirds at-sea. Since the early 90s, when the first seabirds were tracked (Jouventin & Weimerskirch, 1990; Prince et al., 1992), major technological improvements have made tracking devices smaller, more accurate, and more affordable, which diversified their applications and promoted an increase in the number of species tracked (Hays et al., 2016). Similarly, satellite remote sensing has been improved over the last two decades in order to sense relevant oceanographic parameters, such as sea surface temperature (SST) or chlorophyll concentration (McClain, 2009; Blondeau-Patissier et al., 2014). Taken together, tracks of marine animals and oceanographic variables derived from satellite imagery provide exceptional opportunities to understand the functional mechanisms of marine ecosystems (Wakefield, Phillips & Matthiopoulos, 2009). In seabird research the simultaneous use of tracking devices and satellite imagery has escalated since the early 2000s (Tremblay et al., 2009). The most recent studies have taken advantage of the increased spatial and temporal resolution of tracking devices and satellite sensors to identify behavioural responses of seabirds to food patches (e.g., Paiva et al., 2010a; Sabarros et al., 2014; Poli et al., 2017). But while the tools necessary to understand how seabirds use their sea environment are now available, large oceanic regions, particularly in the tropics, remain poorly studied (but see Catry et al., 2009b; Kappes et al., 2011; Le Corre et al., 2012; Legrand et al., 2016; Zajkova, Militao & Gonzalez-Solis, 2017 as examples of tracking studies with tropical seabirds).

The vast majority of seabird tracking studies have been conducted in temperate and polar regions. Those have generally shown that seabirds concentrate their foraging in areas of high ocean productivity, typically characterized by high abundance of phytoplankton and low SST (e.g., Pinaud & Weimerskirch, 2005; Suryan et al., 2006; Paiva et al., 2010b). Productive areas normally match with regions of upwelling, where nutrient-rich water rise to the surface, in consequence of specific sea bottom and current profiles, and supports the development of phytoplankton (Mann & Lazier, 2006). Seabirds repeatedly commute to these areas from their breeding colonies (Weimerskirch, 2007; Wakefield et al., 2015), typically travelling in a linear path and constant speed between the breeding colony and the feeding areas where their path becomes highly tortuous and slow (Weimerskirch, 2007). In contrast, tropical seabirds tend to show looping movements, where feeding events are sparsely distributed along their loop shaped paths, and they normally present low fidelity to feeding areas (Weimerskirch, 2007). Several authors have argued that while polar, temperate and subtropical seabirds feed on areas with predictable productivity (e.g., shelf slopes, ice edges, or ocean fronts), tropical seabirds feed to a large extent in association with subsurface predators (large predatory pelagic fish and cetaceans) that force fish schools towards the surface (e.g., Catry et al., 2009b; Jaquemet et al., 2014; Miller et al., 2018).

Tropicbirds are enigmatic seabirds that typically forage solitarily in tropical and subtropical seas (Jaquemet, Le Corre & Weimerskirch, 2004; Spear & Ainley, 2005). They have been traditionally grouped with pelicans, cormorants, gannets, boobies and frigatebirds in the order Pelecaniformes, but recently they were found to be more closely related to the Eurypygiformes, that include the Sunbittern (*Eurypyga helias*) and the Kagu (*Rhynochetos jubatus*), based on whole-genome analyses (Jarvis et al., 2014). This makes them unique among seabirds taxonomically. Ecologically, they share with boobies, gannets and terns the ability of plunge diving, but unlike these species, they avoid foraging in large multi-species flocks (Spear & Ainley, 2005). They are also unusual in that although they fly long distances (comparable to procellariids), they lack the ability to soar (Spear & Ainley, 1997; Mannocci et al., 2014; Campos et al., 2018). This flight behaviour seems to be possible because they rest for long periods on the water between periods of flight (Spear & Ainley, 2005; Mejias et al., 2017). Despite these unusual characteristics, tropicbirds have only been tracked in a few studies (Pennycuick et al., 1990; Le Corre et al., 2012; Soanes et al., 2016; Mejias et al., 2017; Campos et al., 2018), which greatly limits our understanding of their foraging ecology.

In this study we GPS-tracked 15 White-tailed Tropicbirds (*Phaethon lepturus*) at high frequency in order to infer their foraging behaviour at sea. All of the tracked individuals were caught during chick-rearing in Fernando de Noronha, a tropical oceanic Archipelago offshore of the Northeast Brazilian coast. Foraging behaviour of White-tailed Tropicbirds at sea was examined in the light of oceanographic variables derived from high spatial resolution Moderate-resolution Imaging Spectroradiometer (MODIS) imagery. With this approach we aimed to understand how White-tailed Tropicbirds respond behaviourally to gradients of chlorophyll-a, SST, and turbidity, which were shown to be major drivers of foraging habitat use by many seabird species (Henkel, 2006; Tremblay et al., 2009). We hypothesised that White-tailed Tropicbirds will increase their foraging efforts in areas of high primary productivity, which is expected to correlate with prey availability.

## Methods

### Study area and data collection

This study was conducted in Fernando de Noronha Archipelago (3.86°S, 32.42°W), 354 km offshore of the Northeast Brazilian coast. The archipelago is composed of 21 islands and islets occupying ca. 26 km^2^, and has been protected by Brazilian legislation as a marine national park since 1988. The islands hold large concentration of tropical seabirds, including a breeding population of 100 to 300 of White-tailed Tropicbirds (Leal et al., 2016). The climate is tropical with two marked seasons, the rainy season from January to August and the dry season from September to December. The average annual temperature is 27 °C and the rainfall is 1,400 mm (Leal et al., 2016). The region is influenced by two main oceanic currents, the near surface westward central branch of the South Equatorial Current and the deeper eastward South Equatorial Undercurrent (Tchamabi et al., 2017). The seawater is considered oligotrophic, where phytoplankton productivity is limited by low nutrient concentrations (de Souza et al., 2013). Surface salinity ranges between 35.0 and 37.0‰ (Leal et al., 2016).

**Figure 1:**
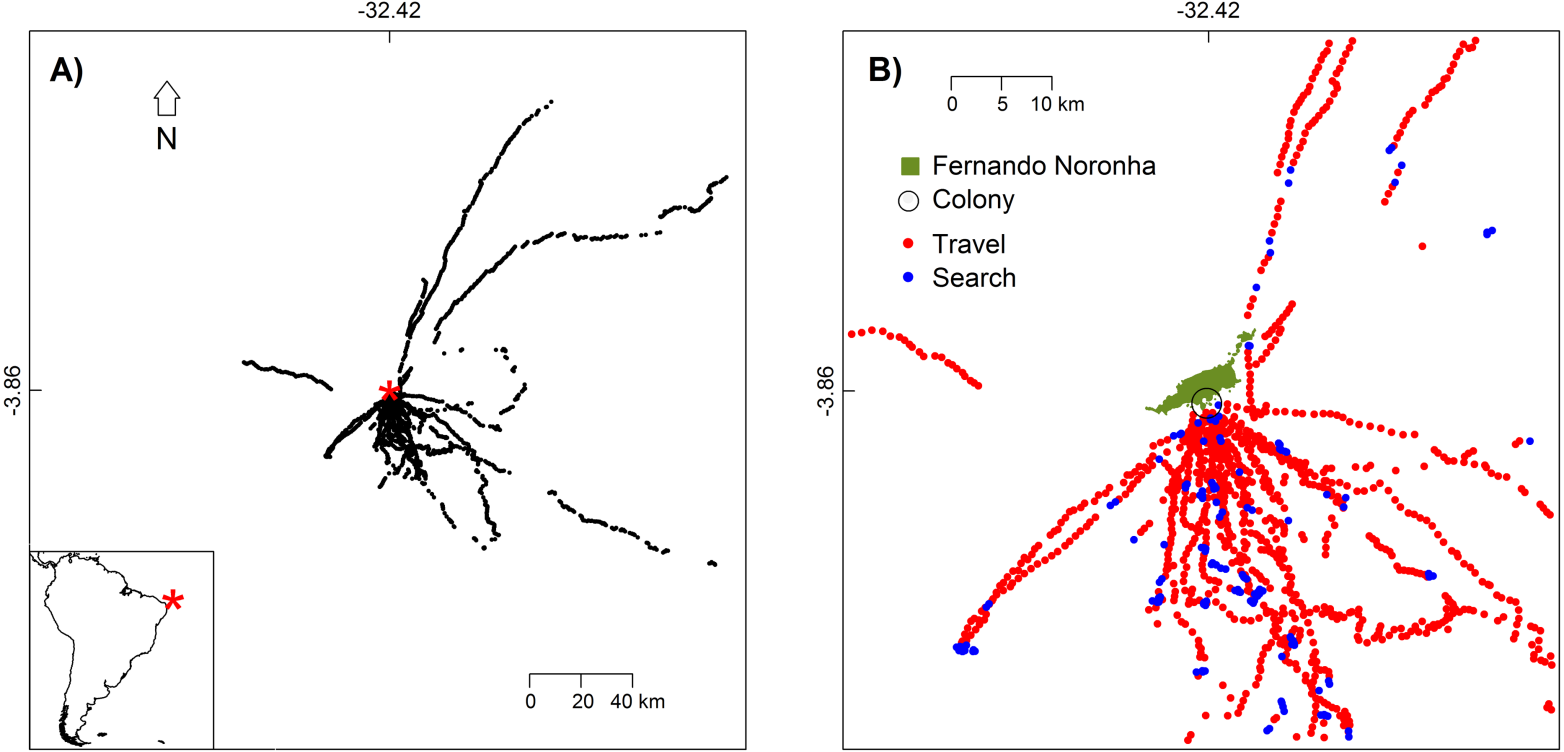
White-tailed Tropicbirds behaviour at sea classified from First-Passage Time analysis. (A) Location of the colony (red asterisk) and locations where behaviour was classified (black dots). (B) Flight behaviour classified as travel and search. Only the part of the study area with higher bird use is shown.

Tracked White-tailed Tropicbirds were captured during chick-rearing (chicks of 1 to 3 weeks old) in Morro do Chapéu islet (Fig. 1), which holds the largest breeding colony of this species in the whole archipelago (Leal et al., 2016). The 15 tagged White-tailed Tropicbirds were captured by hand in the nest at dawn (4 to 4:30 am), before they leave for the sea, between August 28th to October 16th 2015. Birds weighted between 315 to 435 g (average 355 g). The data loggers (5 g including battery, Gipsy 4 GPS recorders, Technosmart, Italy) were waterproofed with heat shrink tubing (increasing their weight to 10-15 g) and were attached to the bases of the four central tail feathers with duct tape. The handling of the animals took less than 10 min, after which they were immediately released.

The experimental procedures of this study, including bird trapping and the GPS tagging, were approved by the Instituto Chico Mendes de Conservação da Biodiversidade (ICMBio) through the license SISBIO 27714-4.

### Data analysis

GPS tracks of White-tailed Tropicbirds flying over the sea were selected from the original dataset. This excluded track segments at the breeding colony or flying over the islands. Some tracks were not round-trip, because the data logger battery ended before the animal returned to the colony. Average GPS recording duration was 8 h and 40 min. GPS data loggers functioned at different rates (from one to six fixes per minute), but to use a consistent dataset we reduced all tracks to the same resolution of 1 fix per minute. Fixes where speed was less than 10 km/h were considered indicative of bird sitting on water (Weimerskirch et al., 2002; Weimerskirch et al., 2005; Weimerskirch, Le Corre & Bost, 2008; Zavalaga et al., 2012; Cecere, Gaibani & Imperio, 2014) and were removed from further analysis.

In order to classify foraging behaviour of White-tailed Tropicbirds at sea, we used First-Passage Time (FPT) analysis following Fauchald & Tveraa (2003), which we implemented in R (R Core Team, 2016) with the function fpt of the package adehabitatLT (Calenge, 2006). FPT is defined as the time required to cross a circle with a given radius, and the circle radius associated with the peak log(variance) of FPT is defined as the scale of Area-Restricted Search (ARS; Fauchald & Tveraa, 2003). We plotted FPT log(variance) for radii ranging from 5 to 1,000 m for each track and determined the scale at which FPT log(variance) peaked. For latter analysis we used the scale of 434 m, corresponding to the median of scales obtained for individual tracks. Bird behaviour was classified into “travel” or “search” based on the histogram of FPT values shown in Fig. S1. The vast majority of observations had FPT values concentrated between 50 and 300 s, while the remaining were distributed in low frequencies among a wide range of FPT values higher than 300 s (Fig. S1). The latter observations were classified as “search” as it is expected that foraging effort demands high residency time. Among the former observations, only a half with the lowest FPT values were classified as “travel” (Fig. S1). The remaining observations, with intermediate FPT values, were excluded from further analysis (Fig. S1). We excluded these intermediate observations because there was no clear division in FPT values, but we needed distinct flight behaviours that could be interpreted against oceanographic variables.

White-tailed Tropicbirds behaviour at GPS locations was examined in the light of oceanographic variables derived from MODIS, made available by the NASA’s OceanColor Web (http://oceancolor.gsfc.nasa.gov). We used the following variables: (1) Turbidity—The Diffuse Attenuation Coefficient at the 490 nm wavelength (commonly referred as Kd(490)) served as a proxy for water turbidity (Shi & Wang, 2010; Loptien & Meier, 2011; Stramska & Swirgon, 2014). The Kd(490) specifically reflects the diffuse attenuation for downwelling irradiance at 490 nm in m^−1^ (see O’Reilly et al., 2000 for details). (2) Chlorophyll-a - Near-surface concentration of chlorophyll-a in mg m^−3^, inferred from remote sensing reflectance in the blue-to-green region of the visible spectrum (see Hu, Lee & Franz, 2012 for details). (3) SST—Sea surface temperature in °C inferred from the 11 µm and 12 µm long wave infrared bands (see Kilpatrick et al., 2015 for details). Images made available at ca. 0.009 decimal degrees (1 km) spatial resolution were resampled to 0.05 decimal degrees (ca. 5.57 km) in order to reduce the number of pixels with no data due to cloud cover. We related bird behaviour of each tracking day to images of oceanographic variables obtained in the corresponding day and the day before (values of both days were averaged). We used images from the day before because it is likely that White-tailed Tropicbirds decide their route using recent foraging experience. In fact, some individuals tracked in following days repeated sections of their routes, while there was no route overlap in tracks recorded with greater time-separation. In addition, we standardized the original values of oceanographic variables among the different tracking days. This was necessary because the range of values of the oceanographic variables within the area accessible to the birds varied considerably between tracking days. The standardization was done by ranking the values of the images of the oceanographic variables in a scale varying from 0 to 20. All images were cut to the same geographic range, set by the longest track recorded (range: 5.1134° to 2.6054°S in latitude and 33.6806° to 31.1726°W in longitude).

The effects of oceanographic variables on the behaviour of White-tailed Tropicbirds at GPS locations were modelled with binomial Generalized Linear Mixed Model (GLMM), using the function glmer of the R package lme4 (Bates et al., 2016). The response variable was assigned as 1 for the observations classified as “search” and 0 for those classified as “travel”. The oceanographic variables were included in the model as fixed factors and bird identity as random factor. Correlations between fixed effects were low (turbidity vs chlorophyll-a: *r* = 0.22; turbidity vs SST: *r* = 0.14; chlorophyll-a vs SST: *r* = 0.26). Model goodness-of-fit was evaluated through marginal *R*^2^ (variance explained by the fixed effects) and the conditional *R*^2^ (the variance explained by the fixed and random effects) following Nakagawa & Schielzeth (2013).

## Results

We tracked 15 different White-tailed Tropicbirds during one to four trips each. Birds showed higher concentration of movements between the S and SE directions (Fig. 1). Our tracking dataset included 6671 GPS fixes, from which 1792 were used for the classification of bird behaviour.

In general, behaviours classified as “travel” and those classified as “search” were not segregated spatially (Fig. 1B), meaning that the White-tailed Tropicbirds search for food as soon as they leave the breeding colony and all along their route. This is also in agreement with their general route pattern of looping foraging trips rather than commuting foraging trips (Fig. S2).

The oceanographic conditions studied here, turbidity, Chlorophyll-a, and SST, varied considerably during the tracking sampling period (average Pearson’s correlation between images available for the sampling period were 0.01, 0.02 and 0.03 for turbidity, Chlorophyll-a, and SST respectively), illustrating a highly unpredictable environment for the White-tailed Tropicbirds. Overall, turbidity ranged from 0.02 to 0.12 m^−1^ (0.03 ± 0.005, mean ± SD), Chlorophyll-a ranged from 0.02 to 0.8 mg m^−3^ (0.12 ± 0.03, mean ± SD), and SST ranged from 9 to 27 °C (24.4 ± 3.2, mean ± SD).

**Figure 2:**
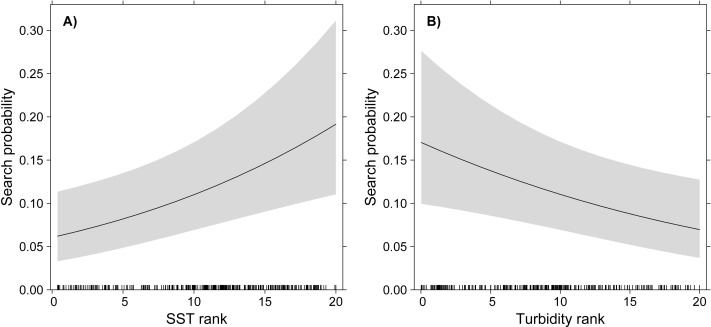
GLMM partial effects of SST (A) and turbidity (B) on the probability of White-tailed Tropicbirds to exhibit search behaviour at sea. The response variable of the model was assigned as 1 for the observations classified as “search” and 0 for those classified as “travel”. The oceanographic variables were included in the model as fixed factors and bird identity as random factor. Shading represents 95% confidence intervals.

The GLMM model showed significant effects of turbidity and SST on the probability of White-tailed Tropicbirds to exhibit search behaviour, but no effect of Chlorophyll-a was observed (Table 1, Fig. 2). The probability of search behaviour increased with the increase of SST (Fig. 2A) and with the decrease of turbidity (Fig. 2B).

**Table 1:** Summary of binomial GLMM testing the effects of oceanographic variables on the probability of White-tailed Tropicbirds to exhibit search behaviour at sea. The response variable was assigned as 1 for the observations classified as “search” and 0 for those classified as “travel”. The oceanographic variables were included in the model as fixed factors and bird identity as random factor. Conditional and marginal R^2^ were calculated following Nakagawa & Schielzeth (2013).

Parameter	Estimate	SE	*Z*	*P*-value	*R*^2^ cond./marg.
Intercept	−2.247	0.384	−5.85	<0.001	0.46/0.09
SST rank	0.065	0.021	3.05	0.002	
Turbidity rank	−0.050	0.020	−2.51	0.012	
Chlorophyll-a rank	−0.002	0.020	−0.11	0.912	

## Discussion

We found that White-tailed Tropicbirds searching for food in oligotrophic waters during breeding show preference for areas with higher SST and lower turbidity (Fig. 2), while no influence of chlorophyll-a was observed (Table 1). Our results also indicate that White-tailed Tropicbirds forage along their foraging trips, showing little spatial segregation between searching and travelling behaviours (Fig. 1B). The general shape of White-tailed Tropicbirds tracks are coherent with the foraging patterns described above, being closer to what is usually defined as looping foraging trips, rather than commuting foraging trips (Fig. S2; Weimerskirch, 2007).

To some extent, these patterns match with general predictions of habitat use by seabirds breeding in tropical oceanic islands, where the sea is largely unproductive and food resources are unpredictable (Weimerskirch, 2007). The sea bottom around tropical oceanic islands is generally deep and flat, with upwelling restricted to scattered seamounts, eddies and frontal zones (Longhurst & Pauly, 1987). In addition, breeding seabirds search for food within a limited range of their colony because their chicks need to be fed frequently (Fauchald, 2009). Thus areas of predictable upwelling are often too far from their reach. The very low temporal correlation in the oceanographic parameters found in our study area during the sampling period supports the idea that feeding conditions around Fernando Noronha are unpredictable. The high conditional *R*^2^ of our model in comparison with the low marginal *R*^2^ (see Table 1) indicates that much of the variation in searching behaviour probability was related to the individual, which may indicate that different individuals rarely find similar foraging conditions due to the unpredictability of the oceanographic conditions. Interestingly, there were several seamounts within the foraging range of the White-tailed Tropicbirds, but they did not use them as feeding areas (Fig. S3). A study in the same region found that even seamounts that reach a few tens of meters below the surface do not disturb the vertical stratification in the euphotic zone (de Souza et al., 2013), thus they are unlikely to create productivity patches usable by the seabirds. In summary, feeding White-tailed Tropicbirds breeding in Fernando de Noronha, seem to be far from areas of predictable productivity, and all the oceanographic parameters measured within their foraging range vary considerably in time and space. This seems to explain why the areas where they feed are scattered and why they develop looping foraging trips (Weimerskirch, 2007).

It may seem counter-intuitive, however, that White-tailed Tropicbirds show preference for warmer and clearer waters as these are associated with low primary productivity (Mann & Lazier, 2006). And this is somewhat supported by the apparent irrelevance of the chlorophyll-a concentration in the choices of foraging areas by the White-tailed Tropicbirds. However, an increasing number of studies in tropical areas have failed to link chlorophyll-a and SST to foraging habitat use of seabirds, or have found negative relationships between bird occurrence and primary productivity (e.g., Vilchis, Ballance & Fiedler, 2006; Jaquemet et al., 2014; Mannocci et al., 2014; Poli et al., 2017). Such results may be explained by spatial mismatches propagated along the trophic chain (Gremillet et al., 2008). White-tailed Tropicbirds, as many other tropical seabirds, prey upon flying fish to a large extent (Stonehouse, 1962; Cherel et al., 2008; Catry et al., 2009a), therefore we should expect a positive relationship between the foraging areas selected by White-tailed Tropicbirds and the distribution of flying fish, and not necessarily the distribution of primary productivity. Interestingly, a recent study using airborne LiDAR and covering a large area (approximately 75,000 km^2^) in the Gulf of Mexico found that abundance of flying fishes increases with SST and decreases with Chlorophyll-a (Churnside et al., 2017). Another recent study confirms the importance of SST explaining the distribution of flying fishes (Lewallen et al., 2017), including one species (*Exocoetus volitans*) that is likely a main prey of White-tailed Tropicbirds in Fernando de Noronha (i.e., this species is a key prey item in the Ascension islands (Stonehouse, 1962), and is abundant in Fernando de Noronha (Monteiro et al., 1998)). In fact, flying fishes are unable to fly at temperatures below 20 °C because their swimming muscles are not able to contract fast enough to take-off (Davenport, 1994), and, presumably, higher temperatures improve flight performance. Similarly, White-tailed Tropicbirds may select clearer waters for other reasons than their productivity. The relevance of water transparency for plunge-divers was recognized long ago by Ainley (1977), who hypothesised that plunge-divers should be distributed towards clearer waters, while pursuit-divers should be more associated with turbid waters. While several studies have confirmed or refuted this hypothesis (Haney & Stone, 1988; Henkel, 2006; Baptist & Leopold, 2010), Haney & Stone (1988) showed from several plunge-divers that the White-tailed Tropicbird was the only species that was significantly more abundant in clearer waters. We believe that water turbidity and prey abundance may interact for the determination of plunge-divers distribution, but it seems logical for us that given equal prey abundance, increased water transparency should help the birds to locate their prey, therefore improving their foraging success.

## Conclusion

Overall, our findings are consistent with previous studies showing that foraging habitat choices of tropical seabirds may not be driven by primary production. While these patterns are scientifically interesting, they also illustrate the true challenge of mapping important foraging areas for tropical seabirds. In this context, the direct use of seabird tracks is still the best approach to identify priority areas for the conservation of tropical seabirds (Le Corre et al., 2012; Soanes et al., 2016). Therefore, additional efforts must be made in order to increase the number of tracking studies in remote areas of the tropical oceans.

##  Supplemental Information

10.7717/peerj.6261/supp-1 Supplemental Information 1 Supplemental figures

## Additional Information and Declarations

### Field Study Permissions

The following information was supplied relating to field study approvals (i.e., approving body and any reference numbers): The field experiments of this study were approved by the Instituto Chico Mendes de Conservação da Biodiversidade (ICMBio) through the license SISBIO 27714-4.

### Data Availability

The following information was supplied regarding data availability: Santos CD, Campos LFAS, Efe MA (2018) Data from: Foraging habitat choice of white-tailed tropicbirds revealed by fine-scale GPS tracking and remote sensing. Movebank Data Repository. DOI 10.5441/001/1.649s6f21.
