# Supplementary material for: Foraging habitat choice of White-tailed Tropicbirds revealed by fine-scale GPS tracking and remote sensing
Source: PeerJ. 2019 Jan 16;7:e6261. doi: 10.7717/peerj.6261 (PMC6339477; doi:10.7717/peerj.6261)
Supplement: Supplemental Information 1 [file peerj-07-6261-s001.pdf]

SUPPLEMENTAL MATERIALS

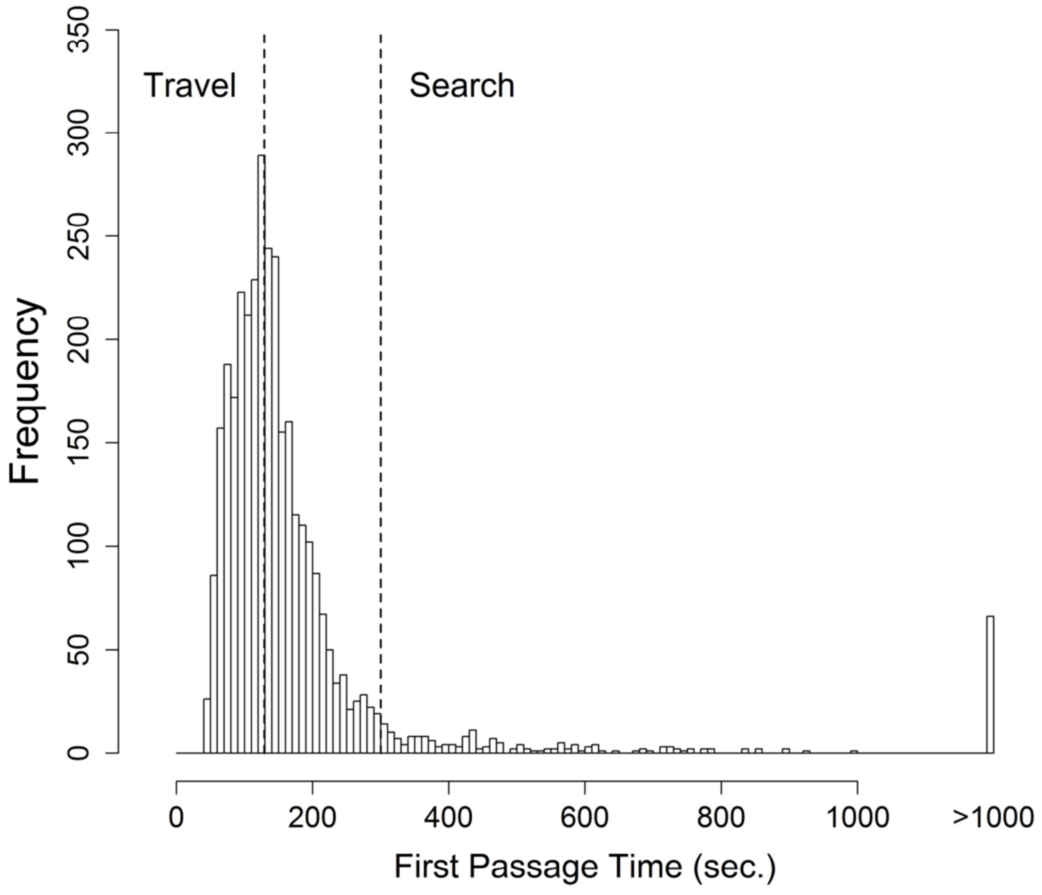

Figure S1. Classification of behaviour of White-tailed Tropicbirds at sea from First-Passage Time (FPT) analysis. Bird behaviour was classified as "search" for FPT values higher than 300 seconds. Among the remaining observations only a half with the lowest FPT values were classified as "travel". Observations with intermediate FPT values were excluded from analysis.

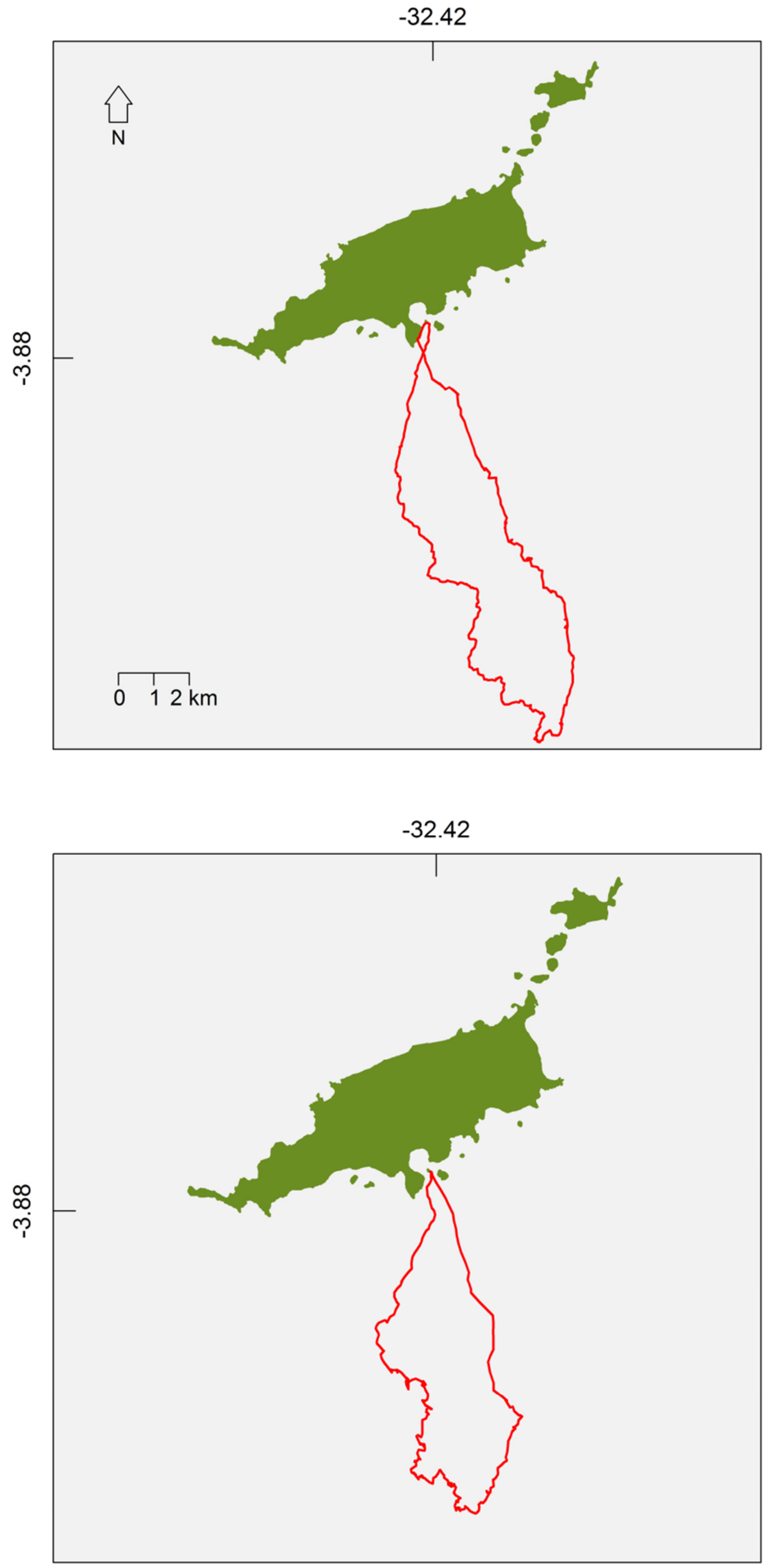

Figure S2. General pattern of White-tailed Tropicbirds foraging trips, illustrated by two tracks recorded with temporal resolution of 0.1 Hz.

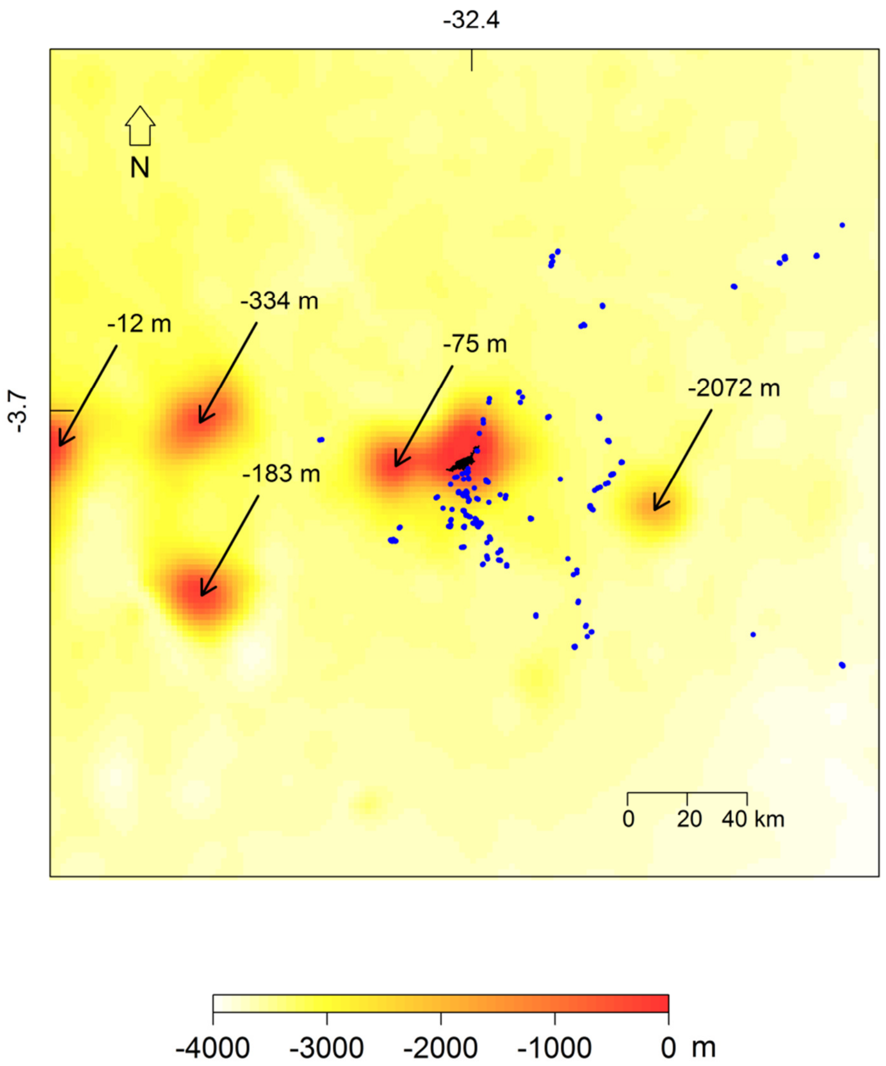

Figure S3. Bathymetry within the range of movements of White-tailed Tropicbirds. Fernando de Noronha archipelago is placed in the image centre in black; blue dots are sampling points where White-tailed Tropicbirds were classified to be searching for food; arrows identify seamounts, with depth above the surface annotated.
